# Smart technique for calculating fault current model parameters using short circuit current measurements

**DOI:** 10.1038/s41598-025-12475-9

**Published:** 2025-08-11

**Authors:** R. A. Mahmoud, O. P. Malik, W. M. Fayek

**Affiliations:** 1 Misr University for Science and Technology (MUST), College of Engineering Science & Technology, Department of Electrical Power and Machines Engineering (PME), 6th of October City, Giza Egypt; 2 Electrical and Software Engineering Department, University of Calgary, Alberta, Canada; 3 Electrical Power and Machines Department, Faculty of Engineering, Helwan University, Helwan, Cairo Egypt

**Keywords:** Fault current model, Decaying time constant, Fault inception angle, Power system angle, Fault locator, Fault recorder, CT saturation, Energy science and technology, Engineering

## Abstract

Precise evaluation of fault current model parameters is an important issue in protection and automation systems. These parameters play a crucial role in selecting protective relay settings, detecting, and compensating saturated CT waveforms, calculating AC and DC components, estimating the sub-transient and transient time periods for the short-circuit current, determining fault locations, and controlling a fault interruption to avoid very fast transients that arise from switching. A new strategy for calculating the fault parameters using short-circuit current model is presented. The short-circuit current data is used to estimate fault inception angle, decay time constant, power system angle and maximum symmetrical AC fault current. The difference concept can be utilized to obtain precise mathematical formulas for evaluating the parameters of the fault current model. This is for efficient implementation of multiple functions that include digital protective relay, fault locator, digital filter, CT saturation detector and compensator. The strategy can be applied offline or in real-time. To verify the developed methodology, comprehensive numerical studies on a power system with real parameters data are presented. The power system is simulated using the Alternative Transient Program (ATP) tool. The algorithm is processed using MATLAB*©* software application. It is examined under variable operating and fault conditions for the system. The quantitative findings indicate that the method has high feasibility, and can achieve reliability, accuracy, and speed in estimating fault current parameters. The results also demonstrate the effectiveness of the proposed algorithm, as well as its robustness with respect to changes in system parameters. Its performance is sustainable as the data window moves, and it is immune to different fault and operational conditions. A key highlight of the proposed approach is the ability to perform many tasks of computer applications in power systems using the accurate calculated parameters for the fault current model.

## Introduction

Electrical power networks are prone to many types of faults and various levels of short-circuit currents^1,2^. In three-phase AC systems, the fault may involve one or more phases and ground, or it may occur only between phases^3,4^. This fault may result in a power interruption, leading to a system’s component breakdown, and in extreme situations, it may cause total failure of the power grid^5^. Thus, it is essential to detect the presence of a fault and isolate the faulty part of the power system as quickly as possible to avoid or reduce damage^6^.

It is desired to perform AC transient analysis, i.e., decompose the short-circuit current into steady state and transient AC components, and the DC component^7^. These components are required for some digital systems used in power systems, such as, digital fault detector, protective relay, fault locator, current transformer (CT) saturation detector and compensator, digital filter, and controlled switching device^8^. It is informed that the short-circuit current may sequentially go through three time spans: sub-transient, transient and steady-state periods^9^. Accurate estimation of the fault current model parameters is crucial for the provision of fundamental and essential information to digital protection and automation systems^10^, as well as fault recorders.

The fault current may be either symmetrical or asymmetrical^11^. The asymmetrical fault current is a function of the X/R ratio of the power system, which is contingent upon the fault position^11^. The greater the X/R percentage, the higher the possible asymmetric current, resulting in larger DC component. The asymmetrical current continues only for a few cycles of the fundamental frequency, after which it shifts to steady-state current. Moreover, it is a function of the symmetrical RMS value of the short-circuit current at fault initiation. Furthermore, system grounding arrangements and impedance affect the asymmetric current. In the case of a single phase-to-ground fault, the greater the grounding impedance, the lower the short-circuit current. The asymmetrical current also relies on the phase angle of the voltage at the time of initiation of the fault. When the fault originates at a point of zero-crossing voltage, this makes the current signal shift from the balanced waveform at normal operating conditions. This is named a DC transient. This is the worst case of asymmetry.

The fully offset asymmetrical short-circuit current is the worst-case considered in the standards for metal-clad switchgear and circuit breakers (CBs) as it imposes the highest mechanical forces on the conductors and their support^12^. However, the conventional fault calculation uses the AC component only, since taking the DC component into consideration requires the solution of many differential equations that describe the dynamic behavior of the whole network^13^. Because the CBs performance can be affected by the DC component besides the AC component, the CBs must be selected with an interruption rating that exceeds the prospective short-circuit current to safely protect the system^14^. Furthermore, in the event of a large DC component, a discrepancy between the real grid current and its measurement might increase due to CT saturation^15,16^. Therefore, accurate estimation of CT secondary current is essential for the protection system to make an appropriate decision^17^.

Another application of fault model parameters is the control of fault interruption^18^. Controlled switching is widely used with CBs to mitigate transients that arise from switching certain loads or fault currents^19^. Shunt capacitor and reactor banks, power transformers and long lines switching are among the most common sources, which cause fast transient of the measured signals. The process of controlled switching aims to achieve a pre-determined target arcing time during fault interruption to reduce electrical wear on the CB^20^. Many techniques have been developed to control switching devices^18–20^, to compensate the distorted CT secondary current for an intended relay operation^10–23^, to assess the decaying DC component^21,23–26^, to identify fault location on the lines^4,27–38^, and/or to provide the fault information to digital fault recorders^39–41^.

Some methods in^42,43^ combine frequency analysis with neural networks or classification with support vector machines (SVM) to observe the chaotic behavior of the current during an arc fault and calculate the current entropy to detect accurately the arc fault.

The conventional digital systems/relays involve the following limitations:

1. The calculated/measured current signals may be unreliable, resulting in incorrect fault diagnosis in the event of severe short-circuit current^10–23^,
2. Stability problems may originate due to delay operating time^10–23^,
3. Relay accuracy is normally less than desired^10–26^,
4. Some approaches need large, sampled signals for fault diagnosis and analysis^1^; therefore, digital signals processing involves large operating time,
5. Some methods have significant impact on the shape and magnitude of voltage and/or current waveforms during fault state^21,23–26^,
6. Some functions require off-line studies^39–41^,
7. Some model-based fault detection approaches generate a residual signal, i.e., difference between the process outputs and their estimates^2^. In the disturbance state, measurement noise, and process transients, the residual signal is non-zero even if there is no fault in the system. Thus, a threshold level is defined to avoid these effects,
8. Complicated mathematical formulas were used to determine the fault current model parameters^6^.

The focus of this paper is the development of a new algorithm to estimate the fault current model parameters using current measurements. In this method, the DC and AC components transients are precisely calculated for use in digital protection and control systems. The proposed method based on short-circuit current model, for AC currents analysis and fault diagnosis of the power system, can be used in several functions that are classified into two groups:

(a) Online and real-time functions, listed as follows:

Digital Protective Relay (DPR),
CT Saturation Detector (SD),
Digital Fault Locator (DFL),
Digital Filter (DF), and
Digital controlled switching device (CB operation control).
(b) Offline functions of computer-based applications, such as:
Digital Fault Recorder (DFR), and
Offline fault model for use in other simulation studies.

The paper is organized as follows: The proposed algorithm is explained in Section "Proposed method", and the description of the power system model is presented in Section "Power system model under investigation". Simulation results are discussed in Section "Simulation results and discussion", and the major features of the suggested technique are outlined in Section "Performance evaluation of the proposed algorithm". Finally, the paper is concluded in Section "Conclusions".

## Proposed method

### Basic concept

The proposed algorithm detects the fault state and estimates the fault current parameters using the fault voltage and current data measured by three-phase voltage and current transformers, respectively, at one terminal of the protected power system element. The algorithm performs two tasks, which are sequentially processed as below:

(1) Detection of the fault inception instant, and.

(2) Calculation of the fault current model parameters using the measured fault voltage and current data.

The algorithm is evaluated as described in the following steps:

(a) Calculate the decay time constant, *τ*_*p*_ (sec).

(b) Estimate the difference angle, *δ* (rad), (where, *δ* = *θ – α*).

where, *θ*: Phase angle of source voltage at fault inception,

*α*: Fault current phase angle (i.e., power system angle).

(c) Compute the maximum symmetrical short circuit current, *I*_*max*_ (in *A*).

(d) Determine the initial current, *I*_*o*_ (in *A*).

(e) Deduce the AC and DC components of fault current; then sum the two components to get the total fault current.

#### Fault current model

Fault current, *i*_*s*_*(t),* can be expressed as a combination of two components^8^. The first component is a periodic component determined by the source voltage and the impedance of the fault circuit. The second component is a DC offset that appears at the fault instant and decays after a few cycles according to the non-defined *L/R* time constant of the power system. The amplitude of the DC component may vary from zero to maximum fault current, depending on the instantaneous value of the voltage at the instant of short-circuit occurrence and on the original power factor of the system^11^. Practically, the voltage and current waveforms that immediately follow faults are never pure sinusoids. This is particularly true for current waveforms, which often contain a relatively large and slowly decaying DC component. When using sinusoidal waveform-based algorithms, care must be taken in dealing with any DC offset components in the current signals, which in practice can be transferred in full to the digital processing stage. The proposed algorithm estimates accurately the DC and sinusoidal AC components using the fault current data measured at one terminal of the faulted element. This is done by calculating the main fault parameters (*I*_*max*_*, θ, α* and *τ*_*p*_) of the short circuit current model. In order to quantify the sinusoidal AC and transient DC values, which can occur during the fault time, consider an RL circuit as a simplified power network. Source voltage, *v*_*s*_*(t)*, is defined by Eq. (1) and phase fault current, *i*_*s*_*(t)*, is modelled according to Eq. (2), as below:

1 $$v{}_{{_{s} }}(t) = V_{\max } [\cos (\omega t + \theta )]$$

2 $$i{}_{{_{s} }}(t) = I_{\max } [\cos (\omega t + \theta - \alpha ) - e^{{ - t/\tau_{p} }} \cos \left( {\theta - \alpha } \right)] + I_{0}^{ - } \times e^{{ - t/\tau_{p} }}$$

3 $$i{}_{{_{s} }}(k) = I_{\max } [\cos (\omega hk + \theta - \alpha ) - e^{{ - hk/\tau_{p} }} \cos \left( {\theta - \alpha } \right)] + I_{0}^{ - } \times e^{{ - hk/\tau_{p} }}$$

4 $$i{}_{{_{s} }}(k) = I_{\max } [\cos (\omega hk + \delta ) - e^{{ - hk/\tau_{p} }} \cos \left( \delta \right)] + I_{0}^{ - } \times e^{{ - hk/\tau_{p} }}$$

where,

*v*_*s*_*(t)*: Instantaneous ‘*S*’ phase source voltage at time instant *t*,

*i*_*s*_*(t)*: Instantaneous ‘*S*’ phase fault current at time instant *t*,

*v*_*s*_*(k)*: Voltage value (*V*) at the sample index *k* of ’*S*’ phase,

*i*_*s*_*(k)*: Current value (*A*) at the sample index *k* of ’*S*’ phase,

*S*: The phase designation *A, B* or* C*,

*V*_*max*_: Peak value of phase source voltage (in *V*),

*I*_*max*_: Peak value of symmetrical phase fault current (in *A*),

I_0_^-^: Instantaneous value (*A*) of pre-fault current (at the fault inception),

*ω*: Angular velocity (*rad/sec*) of power system (*ω* = *2 πf*_*c*_),

*h:* Sampling time interval (s*ec*)*, (h* = *1 / f*_*sp*_*)* and *f*_*sp*_ is the sampling frequency (*Hz*) of the system,

*θ*: Voltage source phase angle (in *rad*) at fault inception,

*α*: Fault current phase angle or the power system angle (in *rad*),

*δ*: Difference angle between the fault inception angle and the power system angle ($\delta$ = *θ – α*), *rad,*

*τ*_*p*_: Time constant (s*ec*) of fault current asymmetrical component, *τ*_*p*_ = *L / R*,

*L*: Source-to-fault inductance (in *Henry*),

*R*: Source-to-fault resistance (in *Ω*),

*T*_*c*_: Cycle period, (*T*_*c*_ = 20 ms) for system frequency of 50 Hz.

*f*_*c*_*:* Fundamental frequency of one periodic cycle, (*f*_*c*_ = 50 Hz),

*T*_*s*_: Sampling time interval, (*T*_*s*_ = *h* = 0.2 ms),

*f*_*sp*_*:* Sampling frequency, (*f*_*sp*_ = 5 kHz), and.

*N*_*s*_*:* The number of samples per cycle for electrical signal (*v*_*s*_(*k*) and* i*_*s*_(*k*)) used in simulation*,* (*N*_*s*_ = *T*_*c*_* / T*_*s*_ or* N*_*s*_ = *f*_*sp*_* / f*_*c*_ = 100 Samples/cycle).

### Fault current parameters estimation

The key parameters (*I*_*max*_*, θ, α* and *τ*_*p*_) of the fault current model are determined based on the difference concept. In the proposed approach, it is assumed that the system angular velocity (*ω*) is based on its immediate pre-fault value. Although, in this article, it is possible to determine accurately the magnitudes of the *AC* and *DC* components, it is impossible to predict the fault location on the sinusoidal cycle. The fault inception voltage angle (*θ*) can either be determined by the process of summing the delta angle and alpha angle (where, *θ* = *δ* + *α*), as will be explained later, or separately from the fault current estimation.

#### Estimation of fault inception angle (θ)

Determination of the instant at which the fault transient starts is important as it is used to obtain the phase voltage fault angle (*θ*), starting the algorithm, and recalling the recorded pre-fault data. The proposed algorithm calculates the fault inception angle (*θ*) of the phase fault current signal by identifying the fault inception instant *(k*_*f*_*)* and the zero-crossing instant *(k*_*z*_*)* of the pre-fault voltage.

##### Determination of the instant of fault inception (*k*_*f*_)

By calculating the first derivative of the measured current signal, it can be observed that the first derivative of the pre-fault current signal is negligible with respect to the first derivative of post-fault current signal. Hence, the first derivative corresponding to the phase currents can be considered as a feature for fault detection. In the proposed method, if the first derivative corresponding to at least one phase current has a value above a threshold value, a fault is ascertained. To determine the fault inception instant for phase current signal, *i*_*s*_*(k)*, the first derivative is defined by a factor *F*_*i*_*,* as:

5 $$F_{i} (k) = |\frac{{i_{s} (k) - i_{s} (k - 1)}}{h}|$$

The proposed technique compares the estimated factor (*F*_*i*_*(k)*) with a pre-determined threshold limit (*F*_*x*_) in order to detect the fault time inception (i.e. *k*_*f*_ = *k*). If the first derivative (*F*_*i*_*(k)*) is greater than the threshold value (*F*_*x*_ = 200% *F*_*m*_), the fault is identified; where *F*_*m*_ is the maximum first derivative obtained from the stored pre-fault cycle of current signal.

##### Determination of zero-crossing for pre-fault voltage (*k*_*z*_)

To determine the zero-crossing instant for phase voltage signal (where, *v*_*s*_*(k)* = *0*), the process is based on multiplication of each two successive samples for the phase voltage data to get a value *v*_*m*_ (where, *v*_*m*_* (k)* = *v*_*s*_* (k-1)* × *v*_*s*_* (k)*). If the value *v*_*m*_*(k)* is negative or zero (i.e., *v*_*m*_*(k)* ≤ *0*), the instant of zero-crossing is verified (i.e., *k*_*z*_ = *k*). This process is performed for the stored pre-fault cycle of the voltage signal. To obtain the fault inception angle (*θ*) of the faulted phase voltage, the following formula can be applied:

6 $$\theta = (k_{f} - k{}_{z}) \times h \times \omega \times (\frac{180}{\pi })(Deg.)$$

After detecting the fault instant, the algorithm can estimate the parameters of the measured short-circuit current. The next stage describes the mathematical formulas for estimating the fault current model parameters.

#### Estimation of DC time constant (*T*_*p*_)

The *L/R* or *X*_*L*_*/(ωR)* ratio identifies the decay time constant (τ_p_), and it strongly influences the current *DC* component. When the system *L/R* ratio is large, it will result in a larger time constant (τ_p_), and slower decay of the *DC* component. In most applications, the *DC* component is completely decayed after just a few cycles. For estimating the equivalent time constant (τ_p_), accurate formulas can be deduced from the fault current model using the difference principle as follows:

(I) Using one-cycle shifting time

7 $$i{}_{{_{s} }}(k) = I_{\max } [\cos (\omega hk + \delta ) - e^{{ - hk/\tau_{p} }} \cos \left( \delta \right)] + I_{0}^{ - } \times e^{{ - hk/\tau_{p} }}$$

8 $$i{}_{{_{s} }}(k + N_{s} ) = I_{\max } [\cos (\omega h(k + N_{s} ) + \delta ) - e^{{ - h(k + N_{s} )/\tau_{p} }} \cos \left( \delta \right)] + I_{0}^{ - } \times e^{{ - h(k + N_{s} )/\tau_{p} }}$$

9 $$i{}_{{_{s} }}(k + m) = I_{\max } [\cos (\omega h(k + m) + \delta ) - e^{{ - h(k + m)/\tau_{p} }} \cos \left( \delta \right)] + I_{0}^{ - } \times e^{{ - h(k + m)/\tau_{p} }}$$

10 $$i{}_{{_{s} }}(k + m + N_{s} ) = I_{\max } [\cos (\omega h(k + m + N_{s} ) + \delta ) - e^{{ - h(k + m + N_{s} )/\tau_{p} }} \cos \left( \delta \right)] + I_{0}^{ - } \times e^{{ - h(k + m + N_{s} )/\tau_{p} }}$$

where,

$$\cos (\omega hk + \delta ) = \cos (\omega h(k + N_{s} ) + \delta )$$

$$\cos (\omega h(k + m) + \delta ) = \cos (\omega h(k + m + N_{s} ) + \delta )$$

11 $$\tau_{p} = [\frac{m \times h}{{\ln (\frac{{i_{s} (k) - i_{s} (k + N_{s} )}}{{i_{s} (k + m) - i_{s} (k + m + N_{s} )}})}}]$$

*τ*_*p*_: is the time constant of the primary system (*τ*_*p*_ = *L/R* = *X*_*L*_*/ωR*). *X*_*L*_ and *R,* respectively, are the reactance and resistance of the primary system to the fault point,

*i*_*s*_*(k)*, *i*_*s*_*(k* + *N*_*s*_*)*, *i*_*s*_*(k* + *m)* and *i*_*s*_*(k* + *m* + *N*_*s*_*):* the four measured values of the secondary current signal *(i*_*s*_*)* for ’*S*’ phase at the instants *(k)*, *(k* + *N*_*s*_*), (k* + *m)* and *(k* + *m* + *N*_*s*_*),* respectively,

##### Remark:

The two samples* i*_*s*_*(k)* and *i*_*s*_*(k* + *N*_*s*_*)* have the same *AC* component. Also the two samples* i*_*s*_*(k* + *m)* and *i*_*s*_*(k* + *m* + *N*_*s*_*)* have the same *AC* component,

*m:* A selected number of samples shifted from the instant *k* (during the fault time), *m* ≤ *N*_*s*_, (in this paper, the *m* is selected as *10* samples).

It is noticed from Eq. (11) that the proposed algorithm identifies the decay time constant (*τ*_*p*_) by using four samples (*i*_*s*_*(k)*, *i*_*s*_*(k* + *N*_*s*_*), i*_*s*_*(k* + *m)* and *i*_*s*_*(k* + *m* + *N*_*s*_*)*) of the actual secondary current measured during the interval that follows directly the fault inception. The above formula needs an execution time between one and two cycles.

(II) Using two-cycle shifting time.

Another mathematical formula for computing the decay time constant (*τ*_*p*_) can be derived when *m* = *N*_*s*_ as:

12 $$\tau_{p} = [\frac{{N_{s} \times h}}{{\ln (\frac{{i_{s} (k) - i_{s} (k + N_{s} )}}{{i_{s} (k + N_{s} ) - i_{s} (k + 2 \times N_{s} )}})}}]$$

where,

*i*_*s*_*(k)*, *i*_*s*_*(k* + *N*_*s*_*)* and *i*_*s*_*(k* + *2* × *N*_*s*_*):* The three measured values of the secondary current signal *(i*_*s*_*)* for ’*S*’ phase at the instants *(k), (k* + *N*_*s*_*) & (k* + *2* × *N*_*s*_*),* respectively, which have the same *AC* component.

It is observed from Eq. (12) that the proposed algorithm estimates the *DC* time constant (*τ*_*p*_) by using three samples (*i*_*s*_* (k)*, *i*_*s*_*(k* + *N*_*s*_*)* and *i*_*s*_* (k* + *2* × *N*_*s*_*)*) of the actual secondary current measured during the interval that follows directly the fault inception; however the latter formula needs an execution time of two cycles.

(III) Using half-cycle shifting time.

Furthermore, to accelerate the computation of the decay time constant (*τ*_*p*_), Eq. (15) can be used:

13 $$i{}_{{_{s} }}(k + N_{s} l2) = I_{\max } [\cos (\omega h(k + N_{s} l2) + \delta ) - e^{{ - h(k + N_{s} l2)/\tau_{p} }} \cos \left( \delta \right)] + I_{0}^{ - } \times e^{{ - h(k + N_{s} l2)/\tau_{p} }}$$

14 $$i{}_{{_{s} }}(k + m + N_{s} l2) = I_{\max } [\cos (\omega h(k + m + N_{s} l2) + \delta ) - e^{{ - h(k + m + N_{s} l2)/\tau_{p} }} \cos \left( \delta \right)] + I_{0}^{ - } \times e^{{ - h(k + m + N_{s} l2)/\tau_{p} }}$$

where,

$$\cos (\omega hk + \delta ) = - \cos (\omega h(k + N_{s} l2) + \delta )$$

$$\cos (\omega h(k + m) + \delta ) = - \cos (\omega h(k + m + N_{s} /2) + \delta )$$

15 $$\tau_{p} = [\frac{m \times h}{{\ln (\frac{{i_{s} (k) + i_{s} (k + N_{s} l2)}}{{i_{s} (k + m) + i_{s} (k + m + N_{s} l2)}})}}]$$

As shown in Eq. (15), to get the primary time constant (τp), the operating time lasts roughly a half-cycle.

#### Calculation of the difference angle (δ) between fault inception angle and power system angle

The mathematical formula for estimating the difference angle $\left(\delta \right)$. is described by eq. (16). This formula is derived from the fault current equation, which combines *AC* and *DC* components. The nominal system angular velocity (*ω)*, the sampling time interval *(h)*, the estimated decay time constant *(τ*_*p*_*)* and three samples of fault current data (*i*_*s*_*(k), i*_*s*_*(k* + *1)* and *i*_*s*_*(k* + *2)*) measured at the instants (*(k), (k* + *1)* and *(k* + *2)*)*,* respectively, are used to calculate accurately the difference angle $\delta$ of the power system*.*

16 $$\delta = \tan^{ - 1} [\frac{{F_{1} - F_{2} \times F_{5} }}{{F_{4} \times F_{5} - F_{3} }}]$$

where,

$$F_{1} = [e^{{ - h/\tau_{p} }} \times \cos (\omega hk) - \cos \left( {\omega h(k + 1)} \right)]$$

$$F_{2} = [e^{{ - h/\tau_{p} }} \times \cos (\omega h(k + 1)) - \cos \left( {\omega h(k + 2)} \right)]$$

$$F_{3} = [\sin (\omega h(k + 1)) - e^{{ - h/\tau_{p} }} \times \sin (\omega hk)$$

$$F_{4} = [\sin (\omega h(k + 2)) - e^{{ - h/\tau_{p} }} \times \sin (\omega h(k + 1))]$$

$$F_{5} = [\frac{{e^{{ - h/\tau_{p} }} \times i_{s} (k) - i_{s} (k + 1)}}{{e^{{ - h/\tau_{p} }} \times i_{s} (k + 1) - i_{s} (k + 2)}}]$$

$\delta$: The calculated difference angle between fault inception angle (*θ*) and power system angle (*α*),

*i*_*s*_*(k)*, *i*_*s*_*(k* + *1)* and *i*_*s*_*(k* + *2):* The measured values of the secondary current signal *(i*_*s*_*)* for ’*S*’ phase at the instants *(k), (k* + *1) & (k* + *2),* respectively.

#### Calculation of maximum current magnitude (I_max_)

To identify the maximum steady state *AC* value of the fault secondary current, Eq. (17) can be applied.

17 $$I_{\max } = [\frac{{e^{{ - h/\tau_{p} }} \times [i_{s} (k) - i_{s} (k + 1)] - [i_{s} (k + 1) - i_{s} (k + 2)]}}{{e^{{ - h/\tau_{p} }} \times [P_{1} - P_{2} ] - [P_{2} - P_{3} ]}}]$$

where,

$$P_{1} = [\cos (\omega hk + \delta ) - e^{{ - hk/\tau_{p} }} \cos \left( \delta \right)]$$

$$P_{2} = [\cos (\omega h(k + 1) + \delta ) - e^{{ - h(k + 1)/\tau_{p} }} \cos \left( \delta \right)]$$

$$P_{3} = [\cos (\omega h(k + 2) + \delta ) - e^{{ - h(k + 2)/\tau_{p} }} \cos \left( \delta \right)]$$

*I*_*max*_: The maximum amplitude of sinusoidal steady state secondary current during the fault,

*τ*_*p*_ $\text{and} \delta$: The estimated values of decay time constant, and the difference angle between the fault inception angle and power system angle, respectively.

*i*_*s*_*(k)*, *i*_*s*_*(k* + *1)* and *i*_*s*_*(k* + *2):* The measured values of the secondary current signal *(i*_*s*_*)* for ’*S*’ phase at the instants *(k), (k* + *1) & (k* + *2),* respectively,

#### Calculation of initial current magnitude (*I*_*0*_)

To evaluate the initial current value (*I*_*0*_), Eq. (18) can be used.

18 $$I_{0} \begin{array}{*{20}c} {} \\ {} \\ \end{array} = \begin{array}{*{20}c} {} \\ {} \\ \end{array} [\frac{{i_{s} (k) - I_{\max } \times P_{1} (k)}}{{e^{{ - hk/\tau_{p} }} }}]\begin{array}{*{20}c} {} & {} & {} \\ \end{array} (18)$$

where, $$P_{1} (k)\begin{array}{*{20}c} {} \\ {} \\ \end{array} = \begin{array}{*{20}c} {} \\ {} \\ \end{array} [\cos (\omega hk + \delta )\begin{array}{*{20}c} {} \\ {} \\ \end{array} - \begin{array}{*{20}c} {} \\ {} \\ \end{array} e^{{ - hk/\tau_{p} }} \cos \left( \delta \right)]$$.

Values of the sampling interval *(h)* and the nominal angular frequency *(ω*) are fixed for any given system. Therefore, the value (*ωh*) appears as constant in the evaluation of the fault current parameters (*δ*,* I*_*max*_ and* I*_*0*_).

### Calculation of AC and DC components

After calculating the fault current parameters*,* the sinusoidal *AC* and the decaying *DC* components are obtained using Eqns. (19) and (20), respectively.

19 $$i{}_{{_{sAC} }}(k) = I_{\max } [\cos (\omega hk + \delta )]$$

20 $$i{}_{{_{sDC} }}(k) = e^{{ - hk/\tau_{p} }} \times [I_{0}^{ - } - I_{\max } \cos \left( \delta \right)]]$$

The evaluated parameters of fault current are computed within a data window range. This window is pre-determined in the processing algorithm. The selected data window size is between one and two cycles. In this study, the selected data window size = 1.1 cycle after fault inception.

### Validation of estimated fault current model

The validation strategy is facilitated using the concept of reverse engineering, which is the scientific process of analyzing and recovering information and knowledge from a digital system. To validate the estimated fault current parameters, the following steps can be executed using the MATLAB M-file:

1. Assume values for the fault current parameters (*ω, h, I*_*max*_*, I*_*0*_*, δ* and *τ*_*p*_),
2. Obtain the instantaneous values of the fault current using the assumed parameters in the fault current model,
3. Apply the proposed mathematical formulas for evaluating the fault current parameters (*I*_*max*_*, I*_*0*_*, δ* and *τ*_*p*_) using the calculated instantaneous values of fault current,
4. Calculate the maximum error percentages between the assumed and estimated fault current model parameters (*I*_*max*_*, I*_*0*_*, δ* and *τ*_*p*_),
5. Repeat the previous steps for various values of the fault current parameters (*I*_*max*_*, I*_*0*_*, δ* and *τ*_*p*_). If the maximum error percentages, calculated for these parameters, are less than 0.1%, the proposed algorithm is considered as a proper numerical method for estimating the fault current parameters.

Extensive results have confirmed that the proposed computational technique is accurate, reliable, and robust for evaluating the fault current parameters utilizing the deduced mathematical formulas.

### Applications of fault current model parameters

**Fig. 1:**
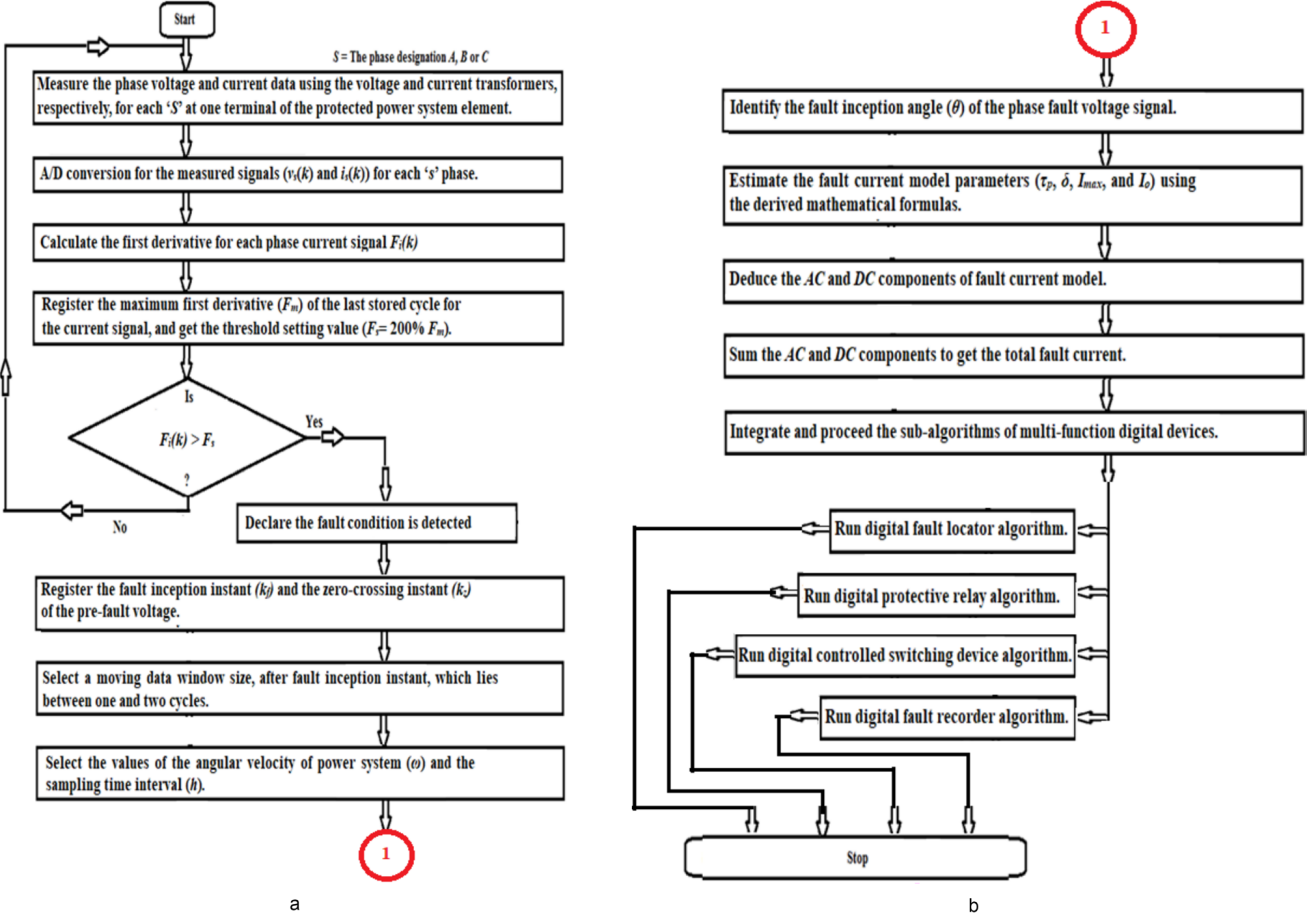
(**a**) Flowchart of the proposed method for computing fault current model parameters. (**b**) Flowchart of the proposed method for computing fault current model parameters.

A flow chart for the computation of fault current model parameters for application in power systems is shown in Fig. 1a,b.

The fault parameters are useful for multi-functions listed below:

1. Digital fault locator,
2. Digital protective relay,
3. Digital controlled switching device (CB operation control),
4. Digital fault recorder,
5. Digital filter,

Steps for each function are presented below.

#### Digital fault locator algorithm

**Fig. 2:**
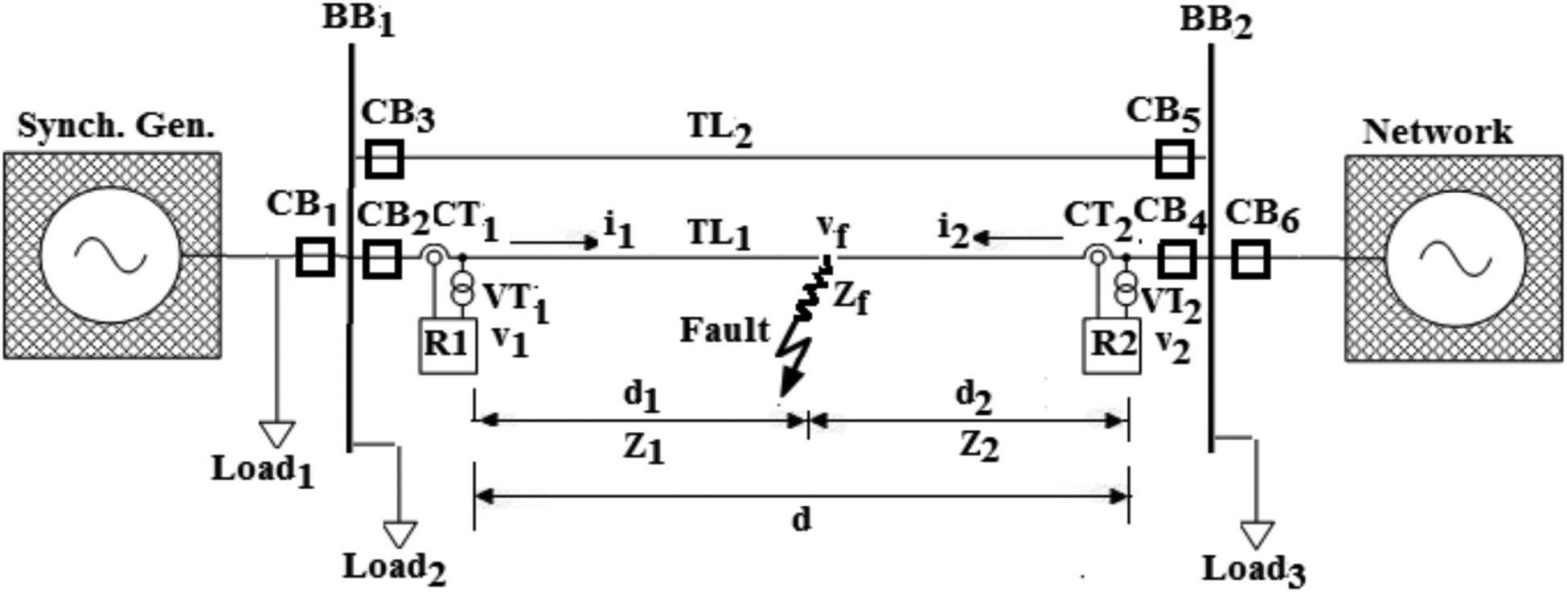
Single line diagram of the power system model under investigation.

To deduce the mathematical formulas used in the proposed algorithm of fault locator, it is mandatory to depict the single line diagram of the power system model under study (as given in Fig. 2).

21 $$Z_{T} = Z_{1} + Z_{2}$$

22 $$Z_{1} = \frac{{V_{1} - V_{f} }}{{I_{1} }}$$

23 $$V_{f} = V_{1} - Z_{1} \times I_{1}$$

24 $$Z_{2} = \frac{{V_{2} - V_{f} }}{{I_{2} }}$$

25 $$V_{f} = V_{2} - Z_{2} \times I_{2}$$

26 $$V_{1} - V_{2} - Z_{1} \times I_{1} + Z_{2} \times I_{2} = 0$$

27 $$V_{f} = Z_{f} \times (I_{1} + I_{2} )$$

28 $$Z_{1} = \frac{{V_{1} - V_{2} + Z_{T} \times I_{2} }}{{(I_{1} + I_{2} )}}$$

29 $$Z_{2} = \frac{{V_{2} - V_{1} + Z_{T} \times I_{1} }}{{(I_{1} + I_{2} )}}$$

where,

*V*_*1*_: Maximum phase voltage for ‘*S’* phase at the sending *TL* end,

*V*_*2*_: Maximum phase voltage for ‘*S’* phase at the receiving *TL* end,

*I*_*1*_: Maximum phase current for ‘*S’* phase at the sending *TL* end,

*I*_*2*_: Maximum phase current for ‘*S’* phase at the receiving *TL* end,

*V*_*f*_*:* Maximum voltage at the fault point,

*I*_*f*_*:* Total short-circuit current obtained from both *TL* sides (*I*_*f*_ = *I*_*1*_ + *I*_*2*_*)*,

*Z*_*T*_: Full impedance of the transmission line extended from *BB*_*1*_ to *BB*_*2*_ (its length is 100 km),

*Z*_*1*:_ Impedance of the faulted *TL* from *BB*_*1*_ to the fault point for ‘*S’* phase,

*Z*_*2*:_ Impedance of the faulted *TL* from *BB*_*2*_ to the fault point for ‘*S’* phase,

*Z*_*f*_: Fault impedance imposed from the faulted point on *TL* to the ground point in case of ground fault or inserted between the two faulted phases in case of phase-to-phase fault,

*d*: Full distance of the transmission line (length 100 km),

*d*_*1*:_ Distance of the located fault on *TL* from *BB*_*1*_ to the fault point,

*d*_*2*:_ Distance of the located fault on *TL* from *BB*_*2*_ to the fault point,

*r*: Resistance per unit length of *TL* (*Ω/km*),

*l*: Inductance per unit length of *TL* (*H/km*),

*z*: Impedance per unit length of *TL* (*Ω/km*),

It is shown that the fault impedance (*Z*_*f*_) has no effect on the accuracy of the TL fault location because *Z*_*1*_ and *Z*_*2*_ are based on the two voltages and currents measured at both TL sending and receiving ends, as well as the total impedance (*Z*_*TL*_) of the protected transmission line.

The fault impedance (*Z*_*f*_) affects the accuracy of the TL fault location if the method uses the three-phase voltage and current measured only at one TL end (i.e., the sending or receiving end). This is because the fault point voltage (*V*_*f*_) is based on the fault impedance (*Z*_*f*_) and both currents of the sending and receiving ends (*I*_*1*_ and *I*_*2*_), which affect the calculated *Z*_*1*_ and *Z*_*2*_.

The proposed algorithm for digital fault locator is described in the following steps:

(1) Obtain the maximum *AC* voltage (*V*_*max*_) and the maximum *AC* current (*I*_*max*_) at both TL ends,

(2) Estimate the fault location (*FL*_*1*_*)* at the TL sending end using the following:

30 $$FL_{1Calculated} = \frac{{Z_{1} }}{{Z_{TL} }} \times 100\% = \frac{{\left| {V_{1\max } } \right|/\left| {I_{1\max } } \right|}}{{Z_{TL} }} \times 100\%$$

(3) Assess the fault location (*FL*_*2*_*)* at the TL receiving end using the following:

31 $$FL_{2Calculated} = \frac{{Z_{2} }}{{Z_{TL} }} \times 100\% = \frac{{\left| {V_{2\max } } \right|/\left| {I_{2\max } } \right|}}{{Z_{TL} }} \times 100\%$$

(4) Calculate the percentage error (*FL*_*Error*_*)* of the fault location via the following mathematical formula:

32 $$FL_{Error} = \frac{{FL_{Calculated} - FL_{Actual} }}{{FL_{Actual} }} \times 100\%$$

where,

*Z*_*F*_: Impedance from the positioning of fault locator to the fault point,

*Z*_*TL:*_ Full *TL* impedance,

*I*_*max*_: Maximum *AC* current,

*V*_*max*_: Maximum *AC* voltage,

*FL*_*Error*_: Percentage error of the fault location,

*FL*_*Calculated*_: Calculated fault location, and.

*FL*_*Actual*_: Actual fault location,

#### Digital protective relay algorithm

(a) Compensation of saturated CT secondary current.

The compensation algorithm for the distorted CT secondary current is processed to:

(1) Identify the fault start instant,

(2) Detect the *CT* saturation inception instant,

(3) Estimate the fault current model parameters (*τ*_*p*_, *δ*, *I*_*max*_, and *I*_*o*_) using the data of the saturation-free portion for *CT* secondary current,

(4) Obtain the *AC* and *DC* components of the fault current,

(5) Add the *AC* and *DC* components to get the total fault current to reconstruct the distorted CT secondary current,

(6) The compensated secondary current of *CT* is useful for sending a blocking action of protective relays (such as differential overcurrent and restricted earth fault relays) in the case of external faults with *CT* saturation,

(7) Moreover, the reconstructed secondary current of *CT* is important for estimating the fault location accurately,

(8) In addition, the compensated secondary current of *CT* is essential for evaluating the time-to-saturation and assessing the severity level of *CT* saturation. The time–to-saturation (*T*_*s*_) is estimated as:

33 $$T_{s} = (k_{s} - k_{f} ) \times h$$

where,

*k*_*f*_: Sample index at which the fault is detected,

*k*_*s*_: Sample index at which the CT saturation starts,

*h:* Sampling time interval (*h* = 0.2 (millisecond).

The *CT* saturation detection factor (*F*_*s*_), which assesses the severity level of *CT* saturation, can be computed as:

34 $$F_{s} = \frac{{CTR \times i_{s} (k) - i_{p} (k)}}{{i_{p} (k)}}$$

(b) The fault current model parameters are useful for digital filter in order to extract the *DC* and *AC* components from the short circuit current and compare them with each other.

#### Controlled switching device algorithm

The fault current model parameters are crucial for phase-controlled closing of a circuit breaker, or for selecting the inception angle for control of CB interruption to isolate the faulted section from the rest of the power system. Overvoltage events can be avoided by controlling the exact instance of the closing of the three phases separately. Controlled closing refers to controlling the conduction point of each CB pole with respect to the voltage phase angle. The CBs used in these applications must be constructed to provide the consistency to successfully repeat the controlled closing operations. Moreover, when switching off the CB to directly cut an inductive load, it is ideally desirable to interrupt the system current during zero-crossing of the current waveform. Nevertheless, practically it is somewhat impossible to maintain the situation.

In normal CB the current interruption may occur at an instant near to the zero-crossing point but not exactly at zero-crossing point of the current signal. As the load is inductive in nature, this sudden interruption of current, causes high *di*_*s*_*(t)/dt* which results in high transient voltage in the power grid. In low or medium voltage power grid, this transient voltage during CB operation may not affect the system performance much, but in Extra and Ultra-High Voltage (EHV and UHV) systems, this is effective. If the contacts separation of CB is not sufficient at the current interruption instant, there may be re-ionization between the CB contacts due to transient overvoltage, hence the arcing may be re-established.

When switching on an inductive load like transformer or reactor, and if the CB closes the circuit near the voltage zero-crossing, there will be high DC components of current. This may saturate the power transformer/reactor core. This causes a high inrush current in the transformer/reactor. When switching on the CB to connect a capacitive load to the system, such as capacitor bank, it is desirable to connect the current path at zero-crossing of system voltage signal. Otherwise, due to sudden change in voltage during switching, high inrush current is originated in the system. This may be followed by over-voltage in the system. The inrush current along with over-voltage impose mechanical and electrical stress. Generally, in CB all three phases open or close nearly at the same instant, but there is 6.6 ms time interval between the zero-crossings of each two adjacent phases of three-phase system operating at a nominal frequency of 50 Hz.

A device, that synchronizes the switching of individual CB poles according to the zero-crossing of the corresponding phase, is known as Phase Synchronizing Device (PSD). Sometimes it is also referred as Controlled Switching Device (CSD). This device is installed at relay and control panel to overcome this transient behavior of voltage and current during switching. The PSD takes three phase voltage signals from three phase Voltage Transformers (VTs) of busbar or load, three phase current signals from thee phase Current Transformers (CTs) of the load, auxiliary contact signal and reference contact signal from the CB, closing and opening command from control switch of the CB installed in control panel. Voltage and current signals from each phase are required to find exact instant of zero-crossing of the waveform of individual phases. CB contact signals are required to calculate the operation time delay of the CB, so that the opening or closing pulse to the breaker can be sent accordingly, to match the interruption and zero-crossing of either current or voltage wave, as per requirement.

#### Digital fault recorder algorithm

The digital fault recorder algorithm combines the above three algorithms of the digital fault locator, protective relay as well as controlled switching device. It can also record the precise parameters of the fault current model.

#### Digital filter algorithm

The digital filter algorithm aims to remove the DC component from the fault current signal and assess the AC component.

## Power system model under investigation

A single line diagram of the power system model under investigation, which is considered for the development of the proposed algorithm, is shown in Fig. 2. The model is composed a of double-circuit, 19.57 kV, 100 km three-phase transmission line (TL*)* extending between a synchronous machine and a connected electrical network, and three loads. The TL is represented by distributed parameters and the frequency dependence of the line parameters is considered. The components of the power system model, simulated by ATP software, are based on realistic parameters given in Table 1, as outlined in the published paper^44^. To validate the proposed approach, extensive simulation studies have been carried out under variable operating and fault conditions using Alternative Transient Program (ATP); and MATLAB software is used for processing the mathematical formulas of fault current parameters.

**Table 1:** Parameters’ data of the power system components.

Power System Parameter	Data
Synchronous Generator (Sending end):	
Rated Volt-ampere	320 MVA
Rated line voltage	19.57 kV
Rated frequency	50 Hz
Number of poles	2
Neutral grounding impedance (*R*_*n*_)	0.00073 Ω
Power Network (Receiving end):	
Nominal line voltage	19.57 kV (1pu)
Voltage phasor angle phase	0^0^
Nominal frequency	50 Hz
Volt-ampere short circuit	1 GVA ( *i*_*s.c*_ = 50 kA)
Transmission Lines (*TL*_*1*_ &*TL*_*2*_):	
Positive sequence *R*	0.0217 Ω /km
Zero sequence *R*	0.247 Ω /km
Positive sequence *XL*	0.302 Ω /km
Zero sequence *XL*	0.91 Ω /km
Positive sequence 1/*Xc*	3.96 µƱ /km
Zero sequence 1/*Xc*	2.94 µƱ /km
Transmission line long (*Km*)	100 km
Aux. Load (load 1):	
Load 1 Volt-ampere	30 MVA at *PF* = 0.85 lag
Aux. Load (load 2):	
Load 2 Volt-ampere	260 MVA at *PF* = 0.85 lag
Aux. Load (load 3):	
Load 3 Volt-ampere	260 MVA at *PF* = 0.85 lag
Current Transformers (*CT*_*1*_ &*CT*_*2*_):	
*CTR* *R*_*CT*_	12,000 A/ 1A 4.9 Ω
*Rated burden*	30 VA
*R*_*burden*_	0.5 Ω
*R*_*lead*_	0.2 Ω
Class	5p20
Voltage transformers (*VT*_*1*_ &*VT*_*2*_):	
*VTR*	19.57 kV/ 100 V
*Rated burden*	30 VA

## Simulation results and discussion

In the proposed approach, samples of three phase currents are taken at the sending end of one transmission line (i.e., at busbar BB_1_). The measured samples are used to evaluate the fault current parameters of the three phase currents. For each case study, the fault data has been generated using ATP program with sampling interval of 0.2 ms, and the selected data window size is 1.1 cycle. The full simulation time is 0.2 s (i.e., the total number of samples *N*_*sim*_ = 1000). The fault data is utilized with MATLAB software that processes the derived formulas for calculating the parameters of fault current model. Extensive simulation case studies are performed to evaluate the algorithm performance under various faults and operating conditions of the power system.

Different scenarios are conducted to validate the performance of the proposed algorithm under the impact of the following instances: (1) Various fault distances from the relay location, (2) Different fault resistances, (3) Different fault types, including balanced, unbalanced, grounded, and ungrounded faults, (4) Different fault inception angles, (5) Different unbalanced/balanced load variations, (6) Different load angles, and (7) Different severity levels of CT saturation. In this study, the fault location is at a point *F* on the simulated power system with distance *d*_*1*_ from the relaying location (i.e., at busbar BB_1_). Also, it is assumed that the line is loaded before the fault initiation time. The pre-fault operating conditions of the simulated power system are given in Table 2. For each case study, *δ*_*1*_ = *30°*, *δ*_*2*_ = *0°*, *F*_*1Operated*_ = *F*_*2Operated*_ = *50 Hz* and* V*_*1Max*_ = *V*_*2Max*_ = *16.063 kV.*

**Table 2:** The pre-fault operating conditions of the simulated power system for each case study.

Electrical component (operating condition)	Data
Operating peak phase voltage of synchronous generator	16.063 kV
Operating peak phase voltage of Electrical power network	16.063 kV
F_1operated_ of synchronous generator	50 Hz
F_2operated_ of electrical power network	50 Hz
Synchronous generator operating power angle (*δ*_*1*_)	30^o^
Electrical power network operating power angle (*δ*_*2*_)	0^o^
Electrical Load 1	10.85 + j 6.72 Ω
Electrical Load 2	1.25 + j 0.75 Ω
Electrical Load 3	1.25 + j 0.75 Ω
CB_1_, CB_2_, CB_3_, CB_4_ , CB_5_ and CB_6_ status	Close

### Case study 1: response of digital fault recorder algorithm

To demonstrate the algorithm effectiveness, various faults have been simulated under different operating and fault situations. The simulation results of the suggested technique are tabulated in Table 3. As seen in the table, the obtained results represent fault detection at sample index corresponding to *k*_*f*_, decay time constant (*τ*_*p*_), calculated angle delta (*δ* = *θ – α*) and maximum fault current (*I*_*max*_). The generated results (for various operating and fault conditions) of the proposed algorithm have confirmed the following information:

**Table 3:** Fault states and the output results of the proposed algorithm.

Fault states					The output results of the proposed algorithm			
Fault Type	Fault Location (*FL*)	Fault Inception Time (*Sec*)	Fault Resistance (*R*_*f*_) (Ω)	Fault Reactance (*XL*_*f*_) (*Ω*)	Fault Inception instant (*k*_*f*_)	Time constant (*τ*_*p*_) (*Sec*)	*δ* = *θ – α* (*Rad*)	Max. fault current (*I*_*max*_) (*A*)
*SLG*	25%	0.103	0	0	515	0.0449	1.0352	947.323
				1		0.0489	1.0302	923.503
				2		0.0530	1.0245	882.649
				3		0.0573	1.0181	871.379
				5		0.0666	1.0040	818.256
				10		0.0845	0.9640	688.680
				15		0.1053	0.9214	620.361
				20		0.1270	0.8783	586.420
				30		0.1760	0.7947	472.295
				40		0.2412	0.7156	446.045
				50		0.3561	0.6425	369.401
		0.102	0	0	510	0.0446	1.0356	974.794
			1			0.0200	0.9516	973.966
			2			0.0129	0.8697	972.277
			3			0.0095	0.7912	962.522
			4			0.0075	0.7166	944.178
			5			0.0062	0.6467	937.884
		0.100	0	0	500	0.0450	1.0352	974.567
		0.102			510	0.0446	1.0356	974.794
		0.103			515	0.0449	1.0352	947.323
		0.105			525	0.0409	0.9693	742.368
		0.110			550	0.0330	0.7494	633.836
		0.112			560	0.0303	0.6831	508.247
	25%	0.102	0	0	510	0.0446	1.0356	974.794
	50%					0.0444	1.0361	659.02
	75%					0.0443	1.0363	527.925
*SLG*	25%	0.102	0	0	510	0.0446	1.0356	974.794
*3LG*						0.0459	1.0337	973.340
*DLG*						0.0451	1.0368	973.170
*DL*						0.0448	0.5295	930.364

(1) As the fault reactance (*XL*_*f*_) increases, the time constant (*τ*_*p*_) increases due to the increase in the ratio L/R,

(2) As the fault reactance (*XL*_*f*_) increases, the maximum AC current (*I*_*max*_) decreases due to the increase in the impedance to the fault point (*R* + *j(X*_*L*_ + *XL*_*f*_*)*),

(3) As the fault reactance (*XL*_*f*_) increases, the delta angle (*δ*) decreases,

(4) As the fault resistance (*R*_*f*_) increases, the time constant (*τ*_*p*_) decreases due to the decrease in the ratio L/R,

(5) As the fault resistance (*R*_*f*_) increases, the maximum AC current (*I*_*max*_) decreases due to the increase in the impedance to the fault point (*R* + *R*_*f*_ + *jX*_*L*_),

(6) As the fault resistance (*R*_*f*_) increases, the delta angle (*δ*) decreases,

(7) As the fault location (*FL*) goes further from the beginning of the transmission line, the time constant (*τ*_*p*_) is almost constant since the ratio L/R is nearly fixed,

(8) As the fault location (*FL*) goes further from the beginning of the transmission line, the maximum AC current (*I*_*max*_) decreases due to the increase in the line impedance to the fault point (*R* + *jX*_*L*_),

(9) As the fault location (*FL*) goes further from the beginning of the transmission line, angle delta (*δ*) is almost constant since the ratio *X*_*L*_*/R* is approaching a fixed value,

(10) With variation of fault type, the time constant (*τ*_*p*_) is about constant since the ratio *X*_*L*_*/R* is nearly fixed,

(11) With variation of fault type, the angle delta (*δ*) is roughly constant except *DL* fault, and.

(12) With variation of fault type, the maximum (*I*_*max*_) AC current is approximately constant except *DL* fault.

### Case study 2: response of digital fault locator algorithm

**Fig. 3:**
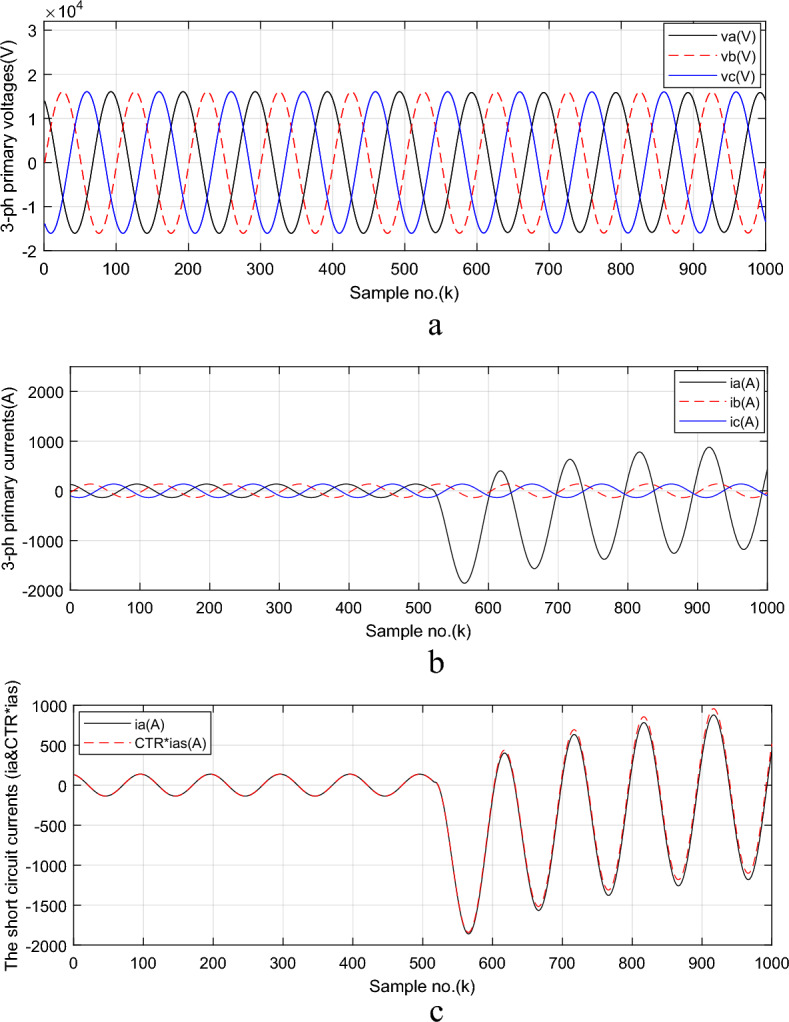
Simulation results for case study 2 (*FL* algorithm).

In this study, the fault current model parameters are used to determine the fault location using the calculated *AC* component for fault current signal. A single line-to-ground (*SLG*) fault located on phase ‘*A’* is applied at point *F*_*1*_ of *TL*_*1*_ with a fault distance *d*_*1*_ = 25 *km* (i.e. *FL*_*Actual*_ = 25% of *TL* length). This distance is from the fault locator positioning at the sending end (i.e., at busbar *BB*_*1*_). In this case, the fault inception time is 0.103 s., the fault resistance and reactance are close to zero (*R*_*f*_ = *XL*_*f*_ = 0 *Ω*), the current transformer residual flux is zero, and the current transformer burden is (0.5 + j0) Ω. The power system angle *(α)* of the primary system is 85.89°. Simulation results of fault locator (*FL*) algorithm are shown in Figs. 3 through 5. The three-phase primary voltage and current signals taken at *TL*_*1*_ sending end are shown in Fig. 3a,b, and the primary and secondary (referred to primary side) fault current signals for ‘*A*’ phase are shown in Fig. 3c. It is observed that the phase ‘*A*’ current signal during the fault interval is higher than the pre-fault currents. They are approximately greater than 6*I*_*n*_; where *I*_*n*_ is the nominal current of *TL*_*1*_. It is noticed that the phase ‘*A*’ fault current signal is accompanied with high asymmetrical component, as shown in Fig. 3c. The fault detection instant (*k*_*f*_) is identified properly using the first derivative of the measured current signals. In this case study, the fault inception instant is *k*_*f*_ = 515 (i.e. *t*_*f*_ = 0.103 s). The produced results have affirmed that the fault inception angle *θ* = 54º (i.e., the closing time switch representing the fault occurrence is at *t*_*f*_ = 0.103 Sec).

**Fig. 4:**
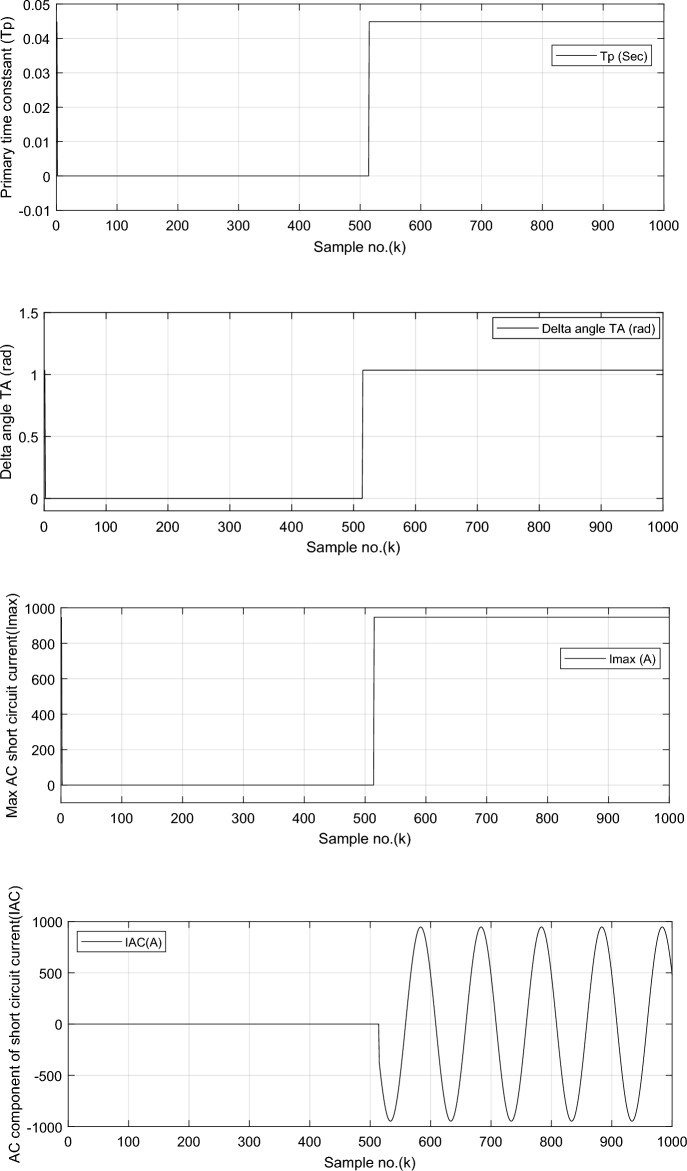
Simulation results for case study 2 (*FL* algorithm: Continued).

The calculated decay time constant (*τ*_*p*_) for phase ‘*A*’ fault current signal, given in Fig. 4a, shows that the decay time constant is close to 0.045 s. The calculated delta angle (*δ*) for phase ‘*A*’ fault current signal, displayed in Fig. 4b, is near 1.0352 rad (i.e., it is corresponding to 59.3°), and the calculated value of maximum *AC* fault current (*I*_*max*_) for phase ‘*A*’, depicted in Fig. 4c, is roughly 947.3 A. The estimated *AC* symmetrical component for ‘*A*’ phase fault current signal is illustrated in Fig. 4d.

**Fig. 5:**
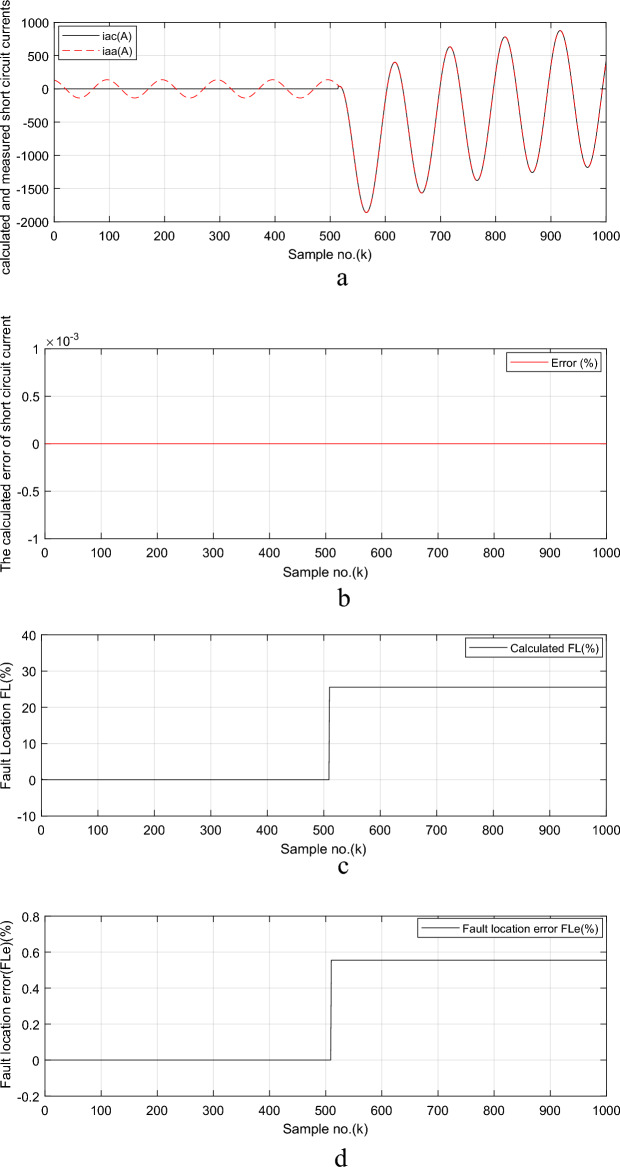
Simulation results for case study 2 (*FL* algorithm: Continued).

The estimated and measured fault current signals (*i*_*ac*_ and *i*_*aa*_, respectively) for phase ‘*A*’ are shown in Fig. 5a. Before and during the fault time, the measured fault current signal *i*_*aa*_ (in red color) is obtained using the ATP program, as shown in Fig. 5a. No estimation is done for the current signal before the fault inception; while, after fault inception, the algorithm starts to calculate only the fault current signal *i*_*ac*_ (in black color) using the MATLAB program application, as depicted in Fig. 5a. After fault inception, Fig. 5a shows the full coincidence between the estimated fault current signal *i*_*ac*_ (in black color) and the measured fault current signal *i*_*aa*_ (in red color) for the ‘*A*’ phase. The percentage error of the estimated fault current signal obtained using the proposed algorithm is given in Fig. 5b. In this study, the obtained fault location (in %) and the *FL* error (in %) of the proposed algorithm are shown in Fig. 5c,d, respectively. It is evident that the estimated distance to *FL* is 25.135 *km* as given in Fig. 5c. This means that *FL*_*Calculated*_ = 25.135% of *TL* length and the *FL* error percentage is close to 0.54%. This confirms the high accuracy of the developed approach. In this study, the transmission line length is 100 km, and the impedance of the full *TL* is *Z*_*TL*_ = 4.34 + j60.4 Ω. Then the value of *Z*_*TL*_ = 60.56 Ω, *I*_*max*_ = 947.3 A,* V*_*max*_ = 14.42 kV and *FL*_*Calculated*_ = (14,420/(60.56*947.3))*100% = 25.135%.

The simulation results reveal that the proposed approach has acceptable performance for a variety of operating and fault situations. An accuracy of 100% is acquired for identifying the faulted TL. The algorithm shows acceptable accuracy even in the case of high fault resistance (i.e., the fault resistance ranges between 50 Ω and 100 Ω). Mean estimation error of the fault location is lower than 0.55% in the *SLG* fault cases with fault resistances (*R*_*f*_) that are lower than 50 Ω, whereas the error of the fault location is greater than 0.55% and lower than 2.2% in the *SLG* fault cases with fault resistances (*R*_*f*_) that lie within the range of 50 Ω and 100 Ω, as shown in Table 4. In this case, the method has used the two voltages and currents measured at both TL sending and receiving sides, beside the full impedance (ZT_L_) of the protected TL. Therefore, the fault resistance (*R*_*f*_) has minor effect on the accuracy of the FL algorithm.

**Table 4:** The FL algorithm results for the *SLG* fault cases with different fault locations.

Fault states					The output results of the proposed FL algorithm				
Fault Type	Fault Location (*FL*)	Fault Inception Time (*Sec*)	Fault Resistance (*R*_*f*_) (Ω)	Fault Reactance (*XL*_*f*_) (*Ω*)	Fault Inception instant (*k*_*f*_)	Estimated FL at SE (*km*)	Error% (at SE)	Estimated FL at RE (*km*)	Error% (at RE)
*SLG*	25% (25 km)	0.103	0	0	515	25.14	0.135	74.86	0.135
			2			24.61	0.393	75.39	0.393
			10			24.58	0.421	75.42	0.421
			20			24.53	0.475	75.47	0.475
			50			25.55	0.545	74.45	0.545
			70			26.38	1.380	73.62	1.380
			80			26.85	1.849	73.15	1.849
			100			27.2	2.195	72.8	2.195

*Note* The DC component is nearly zero in the *SLG* fault cases with high fault resistances that ranges between 10 Ω and 100 Ω.

The proposed method is robust with respect to the variations in TL parameters, making it a proper selection for fault location in TLs for which little information of the TL parameters is available. The proposed algorithm of fault location has the advantage of not requiring prior information about TL parameters. In conclusion, the proposed algorithm provides an accurate, smart, fast, and robust tool for TL fault location.

### Case study 3: response of digital protective relay algorithm with CT saturation condition

*TL* protection requires current transformers for current measurement in the line. During a short circuit, the short circuit current contains a significant DC component that may lead to CT saturation. CT saturation leads to a distorted secondary current. Under such conditions, differential protection systems may result in undesirable tripping; overcurrent and/or distance relays may under-reach or fail to operate in extreme cases. A proper protection scheme should operate even under CT saturation events. To test the appropriateness of the proposed scheme under the state of distorted secondary current, arising out of CT saturation, various simulation cases have been carried out to evaluate the performance of the proposed method in the event of CT saturation. This case reveals the difference between the instantaneous current waveforms of phase ‘*A*’ with and without CT saturation during *SLG* fault. The crucial reasons of CT saturation are low CT ratio (CTR), large CT burden and asymmetry in the current waveform during the transient period. In this case study, the operating conditions of the power system parameters are the same as in case 2 except that the CTR is changed from 12,000/1 to 500/1 and the CT burden is changed from 0.5 + j0 Ω to 50 + j0 Ω. In this case, the fault current model parameters are used to detect and compensate the saturated current signals.

**Fig. 6:**
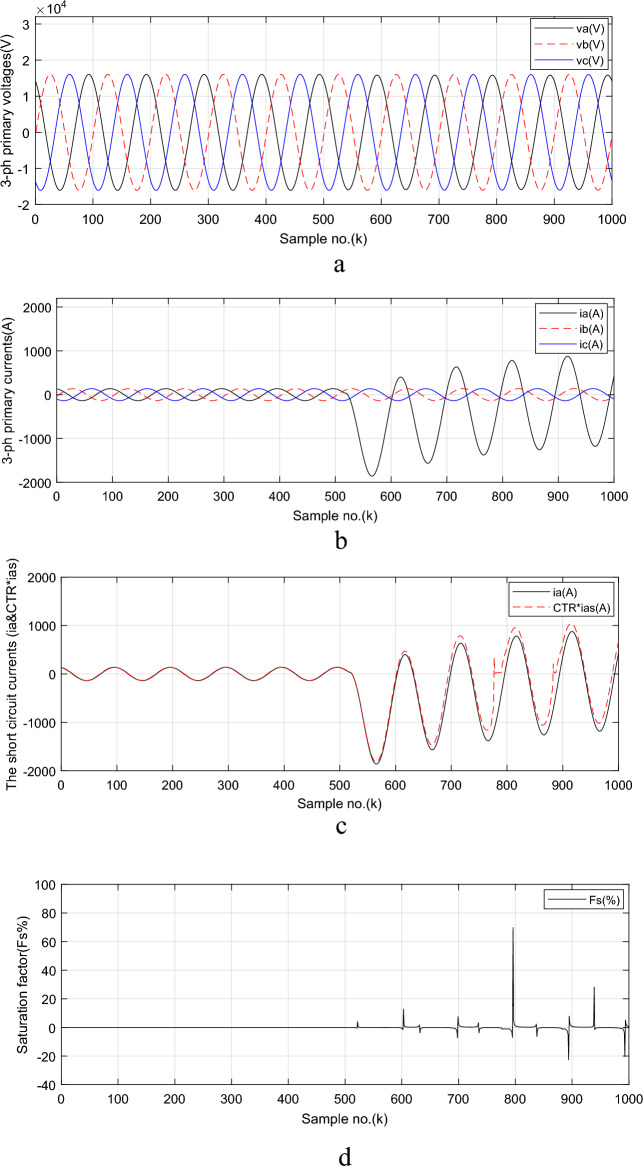
Simulation results for case study 3 (CT saturation condition).

Simulation results for case study 3 are shown in Figs. 6 through 8. The instantaneous primary voltage and current signals measured at *TL*_*1*_ for *BB*_*1*_ side are plotted in Fig. 6a,b and the primary and secondary (referred to primary side) fault current signals for phase ‘*A*’ are shown in Fig. 6c. The phase ‘*A*’ current signal during the fault interval is higher than the pre-fault current. It is greater than 1.2 *I*_*n*_. The fault current signal is accompanied with high DC component. The fault instant is determined by using the first derivative of each measured current signal. Distortion factor (*F*_*s*_), which detects the distortion of secondary phase current signal because of CT saturation, is shown in Fig. 6d.

**Fig. 7:**
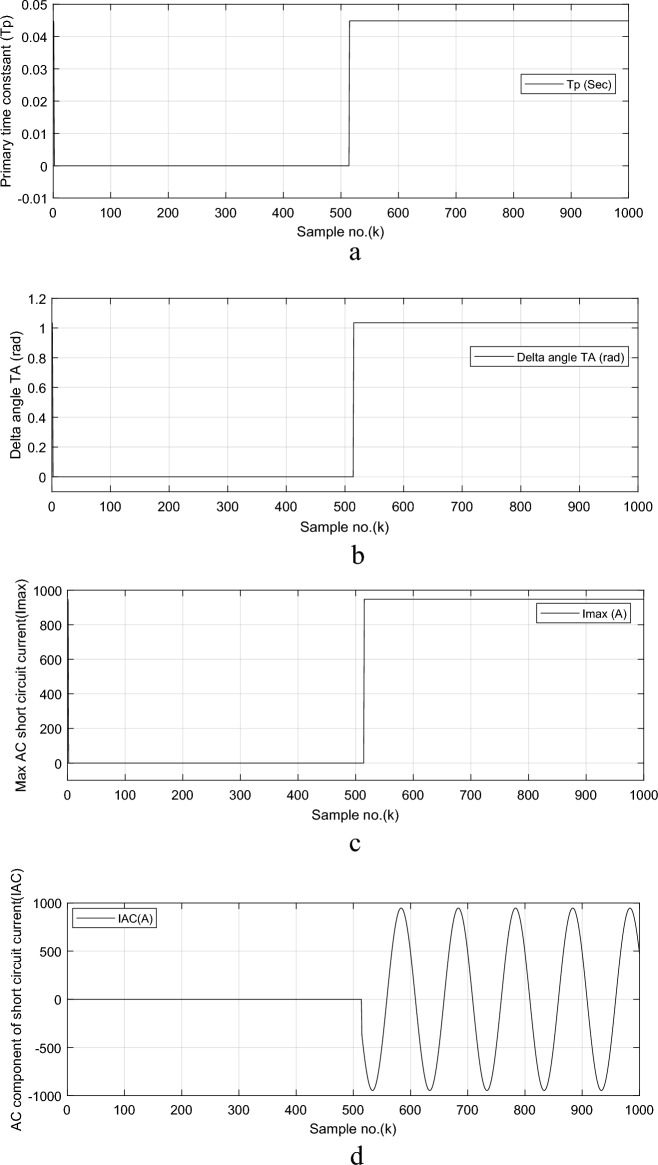
Simulation results for case study 3 (CT saturation condition: Continued).

**Fig. 8:**
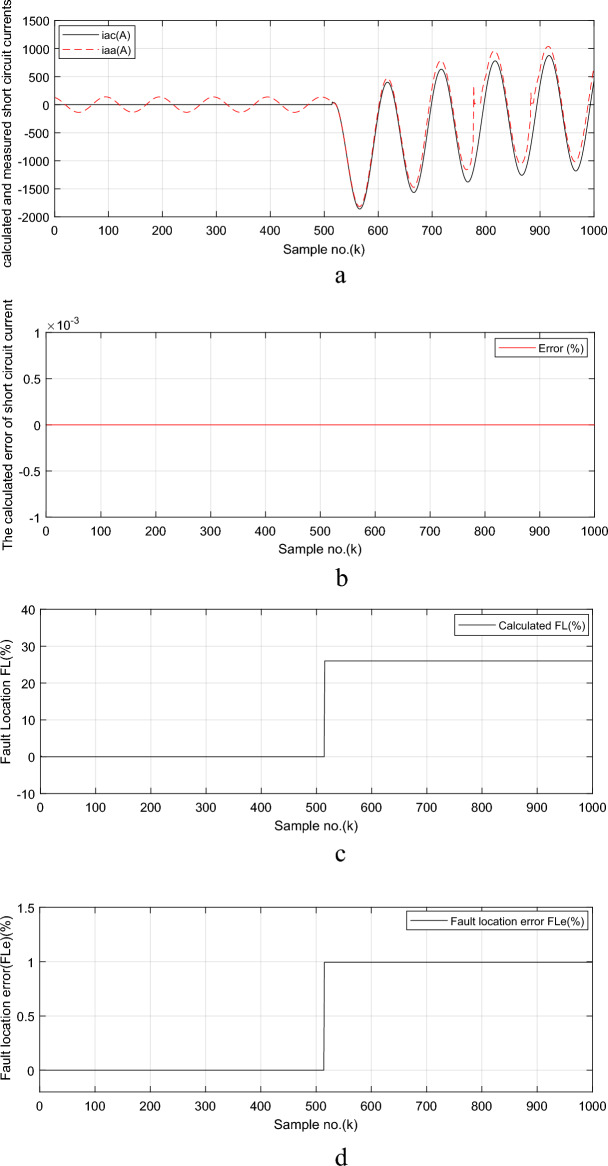
Simulation results for case study 3 (CT saturation condition: Continued).

The calculated decay time constant (*τ*_*p*_) for phase ‘*A*’ fault current signal is shown in Fig. 7a. The time constant is 0.045 s. and the computed delta angle (*δ*) for phase ‘*A*’ fault current signal is depicted in Fig. 7b. Its value is 1.0352 rad (i.e., 59.3°). The maximum AC fault current (*I*_*max*_) for phase ‘*A*’ is illustrated in Fig. 7c. Its magnitude is 947.3 A. The estimated AC component (*I*_*AC*_) for phase ‘*A*’ fault current signal is shown in Fig. 7d. The estimated and measured fault current signal for phase ‘*A*’ is presented in Fig. 8a and the percentage error of the estimated fault current signal for the proposed algorithm is illustrated in Fig. 8b. The estimated fault location (in %) is shown in Fig. 8c, and the *FL* error (in %) of the proposed algorithm for case study 3 is displayed in Fig. 8d. In this case, it is seen that the estimated distance to the *FL* is 25.99 *km* as shown in Fig. 8c. This assures that the technique has high accuracy, where the maximum *FL* error percentage is close to 1%, as developed in Fig. 8d. In this case study, the time–to-saturation (*T*_*s*_), which indicates the severity degree of CT saturation, equals 52.6 ms. This is because with* k*_*f*_ = 515, *k*_*s*_ = 778, and *h* = 0.0002 s, the time–to-saturation (*T*_*s*_) = (778—515) × 0.0002 = 263 × 0.0002 = 0.0526 Sec. Moreover, the CT saturation detection factor (*F*_*s*_) can be evaluated as seen in Fig. 6d.

In this study, ATP and MATLAB software have been used to investigate the proposed approach by carrying out extensive simulation studies considering wide variations in *I*_*max*_*, I*_*0*_*, δ* and* τ*_*p*_. Applicability of the algorithm on a typical power system model with realistic parameters data, for wide variations in operating and fault conditions has ensured the robustness of the proposed technique. Accuracy of the technique has been achieved for all simulation cases, with the maximum error percentage for estimating the fault current parameters found within ± 0.5%. Besides the simulation results have revealed that the proposed approach has correctly identified the fault current model parameters within a time span (starting from the fault inception) of less than two cycles.

In short, after calculating the fault current parameters, the sinusoidal AC and the decaying DC components can be obtained for application in the protection and automation systems of the power grid. These parameters can be integrated into various functions to address different challenges, as described in Table 5.

**Table 5:** The integration between the calculated fault current parameters and various functions to address different challenges.

Function	Integration between the calculated fault current parameters and the function	The algorithm can address the following challenges
**1. Fault detector**	Fault current might be asymmetrical around the time axis. The asymmetrical fault current is a function of the power system X/R ratio at the fault position. As the X/R ratio increases, the possible asymmetry current will be higher, resulting in a larger DC component. The asymmetrical current continues only for a few cycles of the fundamental frequency, after which it shifts to steady-state current. Moreover, it is a function of the symmetrical RMS value of the short-circuit current at the fault initiation. Furthermore, system grounding arrangements and impedance affect the asymmetric current. In the case of a single phase-to-ground fault, the greater the grounding impedance, the lower the short-circuit current. The asymmetrical current also relies on the phase angle of the voltage at the time of initiation to the fault. If the fault originates at a point of zero-crossing voltage, the current waveform will be shifted from the normal balanced waveform. The fully offset asymmetrical short-circuit current is the worst-case. This is named a DC transient	It can be used to discriminate between symmetrical and asymmetrical faults effectively. It can also be applied to estimate the unbalance factor
**2. Digital filter**	The fault current model parameters are useful for the digital filter in order to extract the DC and AC components from the short circuit current and compare them with each other	It can avert the effect of DC components and harmonics
**3. Digital fault locator**	The proposed algorithm for digital fault locator can obtain the maximum AC voltage (V_max_) and the maximum AC current (I_max_) at both ends of the line to specify the fault position	It can determine the fault location accurately (without any errors)
**4. Digital protective relay**	It can run the following functions: (I) Fault detector (II) Digital filter (III) Compensation of saturated CT secondary current: The compensation algorithm for the distorted CT secondary current is processed to: - Estimate the fault current model parameters (*τ*_*p*_, *δ*, *I*_*max*_, and *I*_*o*_) using the data of the saturation-free portion for *CT* secondary current, - Obtain the *AC* and *DC* components of the fault current, - Add the AC and DC components to get the total fault current to reconstruct the distorted CT secondary current, - The compensated secondary current of CT is useful for sending a blocking action of protective relays (such as differential overcurrent and restricted earth fault relays) in the case of external faults with CT saturation, - Moreover, the reconstructed secondary current of CT is important to estimate the fault location accurately	It avoids the malfunction of the differential current relay in the case of external faults with CT saturation
**5. Controlled switching device**	The fault current model parameters are crucial for phase-controlled closing of a circuit breaker (CB), or for selecting the inception angle for control of CB interruption to isolate the faulted section from the rest of the power system	Overvoltage events can be avoided by controlling the exact instance of the closing of the three phases separately
**6. Digital fault recorder**	The digital fault recorder algorithm can combine the functional roles of the digital fault locator, protective relay, and controlled switching device	It can record the precise parameters of the fault current model

Furthermore, the key metrics are quantitative indicators that can be used to evaluate the quality, efficiency, and effectiveness of the algorithm. These metrics are based on different aspects of the algorithm, such as its inputs, outputs, processes, or outcomes. The key metrics used to evaluate the algorithm’s performance are included in Table 6.

**Table 6:** The algorithm verification of the key metrics.

Key metric	Key metric definition	Simulation results from the numerical studies have demonstrated that the algorithm can satisfy the key metric
**1- Speed**	**The speed** evaluates how fast the developed algorithm proceeds or responds to requests	The speed of the proposed algorithm is extremely fast. The data window size can be selected within one cycle of the fundamental frequency (after fault inception)
**2- Accuracy**	**The accuracy** assesses how well the present algorithm achieves the correct results	The accuracy rate of the proposed algorithm is roughly 99.5%. This indicate that it is highly accurate
**3- Robustness**	**The robustness** measures how well the advanced algorithm handles errors, exceptions, or anomalies,	During the sub-transient and transient fault times, the proposed algorithm presents the same quantitative findings of the short-circuit current model parameters at a given fault scenario (for the same power system) using: - Multiple series data windows of the same size, and - Different sizes of moving data windows (half, one, or two cycles) can be used
**4- Reliability**	**The reliability** is a measure of the extent of certitude that it will function correctly. In other words, reliability stands for the inevitability of accurate operation in concert with guarantee against erroneous and inaccurate operation from all extraneous sources Several techniques are used to provide redundant protection, depending on various protective relays selected for use, the requirement for extra functions in the protective relay, and the easiness of implementation. This elevates the reliability of the protection system	The proposed algorithm is considered a reliable numerical method to estimate the fault current model parameters. This is because the maximum error percentage between the assumed and estimated fault current model parameters is less than 0.5% Decay time constant can be obtained using three different formulas. This improves the reliability of the protection algorithm
**5- Scalability**	**The scalability** assesses how well the proposed algorithm handles increasing sizes of data	In this study, the fault data has been generated using ATP program with sampling interval of 0.2 ms. In other sense, the sampling rate is 100 samples per cycle, which denotes a low amount of the sampling

## Performance evaluation of the proposed algorithm

### Protection algorithm attributes

The main characteristics of the proposed algorithm for estimating the fault current model parameters are:

(1) A novel technique to implement efficiently multi-functions that include digital fault detector, protective relay, fault locator, digital filter, CT saturation detector and compensator using fault current model-based approach.

(2) The fault current model parameters play a vital role in the selection of protective relay settings, evaluation of AC and DC components, estimation of the sub-transient and transient periods of short circuit current, detection and compensation of saturated current signals and assessment of fault location. Therefore, the proposed technique is a supplement to digital protective relays.

(3) Fault interruption control is crucial to mitigate transients that arise from switching certain loads or fault currents. This enables the control system to coordinate the trip signal to the circuit breaker to achieve a pre-selected arcing time. This makes the proposed technique a supplement to controlled switching systems.

(4) It is beneficial to calculate the decay time constant of fault current asymmetrical component, then to define the unknown ratio *L/R* (i.e., the ratio between inductance and resistance from a power source to a fault point) to estimate the DC component of the fault current.

(5) It is valuable to obtain the ratio of DC and AC components of fault current signal to be used in the digital filters.

(6) It is useful to detect and compensate the saturated current signals, to evaluate the time-to-saturation and to assess the degree of CT saturation.

(7) It is advantageous to estimate the sub-transient and transient reactance of synchronous generators using the sub-transient and transient components of the short circuit current, respectively.

(8) It is useful to identify the fault location using the AC component of fault current signal. The simulation results show the feasibility of the proposed technique to accurately localize the location of faults in distribution systems.

(9) A sensitivity analysis has been carried out to study the impact of different parameters and operational conditions on the performance of the estimation algorithm, and the simulation results have proved this property.

(10) Comprehensive numerical simulations on a power system with real parameters’ data have demonstrated the effectiveness of the proposed method and provided a proof to this claim.

(11) The proposed algorithm is not only successful in applying a data window principle that contains only the first pre-fault and post-fault cycles; but also, as the data window moves. The algorithm shows full dependability and security, and the performance of the proposed algorithm is sustainable as the data window moves. Based on a comprehensive study, the simulation results have shown that the proposed algorithm is reliable.

(12) The technique can be implemented practically as it is simple.

(13) This technique uses the transient sampling data directly with a short data window size, where the sampling time is 0.2 ms.

(14) The technique has fast response, as the operating time is less than a single cycle.

(15) The technique is independent of the parameters of the instrument transformers, the burden, and the power system elements.

(16) The technique is not affected by the distorted secondary current waveform, under *DC* and *AC* saturation conditions, and operates with the required precision and speed.

(17) The technique has satisfactorily performance under resistive and inductive burdens and under heavy and light *CT* saturation conditions. The simulation results reflect the fact that the fault location, fault inception angle, fault resistance and fault type do not significantly influence the accuracy of the proposed method. This is due to the fault current modelling, in the proposed algorithm, being based on the difference concept.

(18) Three-phase current measurements are sufficient for processing the proposed technique.

(19) The present algorithm can be used in a digital low-pass filter for calculating the DC content of the fault current to reduce its effect on power systems.

### Comparison between the proposed method and other existing techniques

A comparison of the proposed method with the existing methods is given in Table 7.

**Table 7:** A comparison of the protection attributes between the proposed and the existing protection methods.

Ser. No	Item of comparison	Proposed methodology	Existing protection methods
1	Simplicity	Simple	Complex^42,43^
2	Availability	Online	Offline^11,18,40^
3	Speed	Higher Operating time = One cycle = 20 ms	Lower^16,23,25,26^
4	Accuracy	Higher	Lower^9,26^
5	Reliability	Higher	Lower^17^
6	Required data of system parameters	Not Required	Required^40^
7	Functionality Integration	Higher	Lower^40^
8	Detection capability of symmetrical/unsymmetrical faults	Higher	Lower^41^
9	**Functional roles**	Multiple functions (such as fault detection, fault protection, fault location, harmonics filtering, CT saturation detection and compensation, as well as phase-controlled switching of CBs)	Limited functions (such as fault detection and protection)^36–44^
10	**Application fields**	Protection and control systems	Protection systems^21,23,29,30,33,34^

## Conclusions

Exact estimation of fault current model parameters is an important aspect for power system protection and control. An advanced technique to evaluate the model parameters online has been developed, which can be used in numerical protective relays, control schemes, digital fault recorders and fault locators. These parameters encompass the decay time constant, power system angle, fault initiation angle and maximum symmetrical AC short-circuit current. New mathematical expressions for estimating these parameters have been derived from the main model of short-circuit current using the difference principle. Three-phase current measurements are sufficient for processing the proposed methodology to infer the fault parameters. The Alternative Transient Program (ATP) and MATLAB© software applications have been used to testify the effectiveness of the proposed approach on a typical power system model with real parameters’ data.

Performance of the proposed method has been investigated by carrying out numerous simulation studies considering wide variations in fault types, fault resistances, fault inception angles, fault locations, short-circuit current capacity, power flow angles and CT saturation degrees. The applicability of the algorithm to wide variations in operating and fault situations has been verified with high accuracy and robustness of the algorithm. Simulation results have demonstrated the superiority of the proposed method and provided a proof to this claim. Additionally, the outcomes have assured high efficiency and operating speed of the approach in all instances, and immunity to a variety of fault and operational conditions. In other words, the results have proved that the proposed scheme correctly identifies the fault current model parameters within one cycle time following the instant of the fault detection. Moreover, the simulation results have confirmed that the proposed technique is reliable and suitable for diverse operating and fault scenarios. As a consequence, it can be applied as an independent protection scheme or as a supplementary to existing digital protection systems.

Besides, the obtained results have revealed that the proposed CT saturation detection/compensation algorithm has the following features: (I) it is independent of CT characteristics, (II) its performance is not affected by fault current features such as the decay time constant and the DC content present in the current, and (III) it responds rapidly. Therefore, the algorithm can prevent relay malfunction caused by the extent of CT saturation. A key highlight of the suggested scheme is the ability to perform several functions of digital systems in power networks using the accurate parameters evaluated for the fault current model. Some major contributions and benefits of this work include:

(1) The mathematical fault current model of the power system can be established,

(2) It has considerable benefits to other protection schemes, where the algorithm can contribute to the design of fault and CT saturation detectors,

(3) It can integrate the functional roles of the following: digital fault detectors, protective relays, fault locators, digital filters, CT saturation detectors and compensators,

(4) It has the ability to estimate the Short Circuit Capacity (SCC) to measure the AC grid strength,

(5) It can be easily implemented online,

(6) The proposed algorithm is straightforward and invulnerable to factors such as fault classification, fault initiation angle, fault resistance and loading current level,

(7) Both the faulted power transmission line and the fault position can be simultaneously identified,

(8) Acceptable performance has been achieved for both symmetrical and asymmetrical fault types, small fault inception angles and high fault resistance. The fault location accuracy has been improved compared to other schemes. The proposed algorithm provides an accurate, smart, and robust instrument for fault location in parallel-transmission lines,

(9) A sensitivity analysis, examining the impact of different parameters and operational conditions, and evaluating the performance of the estimation algorithm, has also been conducted,

(10) The approach does not require a pre-knowledge of the specifications of the power system elements or the instrument transformers. It is also applicable to power systems with varying voltage ratings,

(11) The approach has been validated on various test systems with different system topologies and network configurations,

(12) According to the simulation outcomes, the proposed algorithm exhibits fast operation, and high dependability in discriminating between normal and fault situations, and.

(13) The short-circuit currents estimated via MATLAB*©* software are almost identical to those measured in ATP simulation program. The results presented in this paper are novel in the sense of mathematical modeling of the short-circuit current.

## Data Availability

All data generated or analysed during this study are included in this published article [and its supplementary information files].

## Abbreviations

*S*: The phase designation *A, B,* or *C*
*N*_*s*_: The number of samples per cycle used in the simulation, (*N*_*s*_ = 100 samples/cycle)
*τ*_*p*_: The decay time constant of the primary system (*τ*_*p*_ = *X*_*L*_*/**ω**R* = *L/R*)
*X*_*L*_: The reactance of the primary system to the fault point
*R*: The resistance of the primary system to the fault point
*i*_*s*_* (k)*: The secondary current signal (*i*_*s*_) at sample index *k* for ’*S*’ phase
*i*_*s*_*(k* + *N*_*s*_*)*: The measured secondary current (*i*_*s*_) at sample index (*k* + *N*_*s*_)
*i*_*s*_* (k* + *m)*: The measured secondary current (*i*_*s*_) at sample index (*k* + *m*)
*i*_*s*_*(k* + *m* + *N*_*s*_*)*: The measured secondary current (*i*_*s*_) at sample index (*k* + *m* + *N*_*s*_)
*i*_*s*_* (k* + *1)*: The measured secondary current signal (*i*_*s*_), at sample (*k* + *1*) for ’*S*’ phase
*i*_*s*_* (k* + *2)*: The measured secondary current signal (*i*_*s*_), at sample (*k* + *2*) for ’*S*’ phase
*M*: A certain shift number of samples, m ≤ N_s_, (the selected *m* is 10 samples in the proposed algorithm)
$\delta$: The difference angle between the fault inception angle (*θ*) and the power system angle (*α*)
*θ*: The fault inception angle
*Α*: The power system angle
*ω*: The angular velocity of the power system (*ω* = *2 **π**f*)
*I*_*max*_: The peak value of the sinusoidal steady state primary current during the fault
*I*_*AC*_*(k)*: The calculated AC component of the current signal (*i*_*s*_) at sample *k* for ‘*S*’ phase
*v*_*s*_*(k)*: The primary voltage at sample *k* for ‘*S*’ phase (at the first side of the *TL*)
*v*_*f*_*(k)*: The voltage of the fault point at sample index *k*
*D*: The full distance of the transmission line (its length is 100 km), *d* = *d*_*1*_ + *d*_*2*_
*d*_*1*_: The distance of the fault located on the *TL* from *BB*_*1*_ to the fault point
*d*_*2*_: The distance of the fault located on the *TL* from *BB*_*2*_ to the fault point
*R*: The resistance per unit length of the *TL* (*Ω/Km*)
*L*: The inductance per unit length of the *TL* (*H/Km*)
*Z*: The impedance per unit length of the *TL* (*Ω/Km*)
*R*_*1*_: The resistance of the faulted *TL* from *BB*_*1*_ to the fault point
*R*_*2*_: The resistance of the faulted *TL* from *BB*_*2*_ to the fault point
*L*_*1*_: The inductance of the faulted *TL* from *BB*_*1*_ to the fault point
*L*_*2*_: The inductance of the faulted *TL* from *BB*_*2*_ to the fault point
*Z*_*t*_: The total impedance of the full length of *TL* from *BB*_*1*_ to *BB*_*2*_*, Z* = *Z*_*1*_ + *Z*_*2*_
*Z*_*1*_: The impedance of the faulted *TL* from *BB*_*1*_ to the fault point
*Z*_*2*_: The impedance of the faulted *TL* from *BB*_*2*_ to the fault point
*Z*_*f*_: The fault impedance imposed from the faulted point on *TL* to the ground point in the case of ground fault or inserted between the two faulted phases in the case of phase fault
*R*_*f*_: The fault resistance imposed from the faulted point on *TL* to the ground point in the case of ground fault or inserted between the two faulted phases in the case of phase fault
*XL*_*f*_: The fault reactance imposed from the faulted point on *TL* to the ground point in the case of ground fault or inserted between the two faulted phases in the case of phase fault
*ATP*: Alternative Transient Program
*t*_*f*_: The fault inception time
*N*_*sim*_: The total number of samples per the full simulation time
*SLGF*: Single Line-to-Ground Fault
*DLGF*: Double Line-to-Ground Fault
*DLF*: Double Line Fault
*3LGF*: Three Line-to-Ground Fault
*AC component*: Alternating current component
*DC component*: Direct current component
*V*_*1Max*_: The peak phase voltage of synchronous generator
*V*_*2Max*_: The peak phase voltage of the electrical network
*F*_*1op*_: The operating frequency of synchronous generator
*F*_*2op*_: The operating frequency of the electrical network
*δ*_*1*_: The operating power angle of synchronous generator
*δ*_*2*_: The operating power angle of the electrical network
*R*_*n*_: Generator grounding resistance through the neutral point
*V*_*n*_: The nominal voltage of the power system
*I*_*n*_: The nominal current of the power system
*R*_*b*_: The current transformer burden
*R*_*CT*_: The current transformer secondary winding resistance
*R*_*lead*_: The lead resistance connected between the current transformer terminals and its burden
*v*_*s*_*(t)*: Instantaneous ‘*S*’ phase source voltage at time instant *t*
*i*_*s*_*(t)*: Instantaneous ‘*S*’ phase fault current at time instant *t*
*V*_*max*_: Peak value of source voltage
*I*_*0*_^*-*^: Instantaneous value of pre-fault current (at fault inception)
*T*_*c*_: The cycle time period, (*T*_*c*_ = 20 *mSec*)
*F*_*c*_: The fundamental frequency of one periodic cycle, (*F*_*c*_ = 50 Hz)
*h*: The sampling time interval, (*h* = *1/ f*_*sp*_ = 0.2 *mSec*)
*f*_*sp*_: The sampling frequency of the system (*f*_*sp*_ = *5** kHz* used in the proposed algorithm)
*k*_*z*_: The zero-crossing instant
*k*_*f*_: The sample index at which the fault occurs (The fault inception instant)
*k*_*s*_: The sample index at which the saturation starts
*F*_*x*_: The pre-determined threshold limit in order to detect the fault time inception. Its value can be determined between 1.1 and 1.2 per unit
*F*_*s*_: The *CT* saturation detection factor, which assesses the severity level of *CT* saturation
*FD*: Fault Detector
*FL*: Fault Locator
*TL*: Transmission Line
*TL*_*1*_: Transmission Line no. 1
*TL*_*2*_: Transmission Line no. 2
*VT*: Voltage Transformer
*VT*_*1*_: The voltage transformer 1 at sending *TL* end
*VT*_*2*_: The voltage transformer 2 at receiving *TL* end
*CT*: Current Transformer
*CT*_*1*_: The current transformer no.1 at sending *TL* end
*CT*_*2*_: The current transformer no.2 at receiving *TL* end
*CTR*: Current Transformer Ratio
*VTR*: Voltage Transformer Ratio
*BB*: Busbar
*BB*_*1*_ and *BB*_*2*_: Busbar no. 1 and 2, respectively
*CB*: Circuit Breaker, *CB*_*1*_*, CB*_*2*_*, CB*_*3*_* CB*_*4*_*, CB*_*5*_*, CB*_*6*_
